# Using objective measures to examine the effect of suspect-filler similarity on eyewitness identification performance

**DOI:** 10.1186/s41235-022-00442-1

**Published:** 2022-10-22

**Authors:** Geoffrey L. McKinley, Daniel J. Peterson

**Affiliations:** Department of Psychology, Skidmore College, 815 N. Broadway, Saratoga Springs, NY 12866 USA

**Keywords:** Lineup construction, Similarity, Multidimensional scaling, Eyewitness memory

## Abstract

When selecting fillers to include in a police lineup, one must consider the level of similarity between the suspect and potential fillers. In order to reduce misidentifications, an innocent suspect should not stand out. Therefore, it is important that the fillers share some degree of similarity. Importantly, increasing suspect-filler similarity too much will render the task too difficult reducing correct identifications of a guilty suspect. Determining how much similarity yields optimal identification performance is the focus of the proposed study. Extant research on lineup construction has provided somewhat mixed results. In part, this is likely because similarity is often defined in relative terms due to the subjective nature of similarity. In the current study, we propose an experiment in which we manipulate suspect-filler similarity via a multidimensional scaling model constructed using objective facial measurements. In doing so, we test the “propitious heterogeneity” and the diagnostic-feature-detection hypotheses which predict an advantage of lineups with low similarity fillers in terms of discriminability.

## Significance statement

When an individual is a witness to a crime, they will often be presented with a lineup of faces to probe their recognition of a police-selected suspect. When constructing these lineups, investigative officers must consider how similar the fillers (i.e., known innocent individuals) are to the suspect. Fillers that are too dissimilar result in a suspect standing out, leading to inflated suspect-identifications. This is problematic because sometimes the suspect that the police have selected is (unfortunately) not the perpetrator of the crime. Such faulty identifications are the leading cause of wrongful imprisonment. On the other hand, when the fillers are too similar to the suspect, the task can be overly difficult and witnesses may fail to identify the (guilty) suspect. Accordingly, in the present study we explored just how similar the fillers and suspect should ideally be to maximize accurate identifications while minimizing false identifications. Traditionally, similarity has been defined subjectively, often by groups of human raters leading to somewhat mixed conclusions regarding prescriptive recommendations of suspect-filler similarity. In the current study, we propose an experiment in which we manipulate this variable via a multidimensional scaling model constructed using facial measurements to provide a more objective means at determining optimal similarity to maximize witness performance.

## Introduction

To date, there have been 387 documented cases litigated by the Innocence Project in which a wrongfully incarcerated individual was later exonerated by DNA evidence (National Registry of Exonerations, 2022). In 256 (66%) of the cases, one or more eyewitnesses erroneously identified the innocent suspect as the perpetrator (National Registry of Exonerations, 2022) rendering it the single greatest contributor for wrongful incarcerations in the U.S. Though eyewitness testimony is an indispensable component of the criminal justice system, this underscores what researchers have argued for decades: eyewitness memory is fallible. Accordingly, it is important that eyewitness memory researchers (a) better understand those circumstances in which witnesses are most likely to make a misidentification, and (b) determine which procedures the criminal justice system can implement to minimize or mitigate the impact of such errors.

These two branches of exploration are commonly referred to as estimator and system variables, respectively. Estimator variables are factors that influence the likelihood a witness makes an accurate identification but (critically) are beyond the criminal justice system’s control. For example, one can intuit that the more time a witness has to study a face the more likely they are to make an accurate identification during a subsequent lineup. This intuition is supported by empirical findings (e.g., Palmer et al., 2013), but it is important to note that encoding time is not controlled by the criminal justice system. Accordingly, beyond simply acknowledging that some witnesses will have a poorer or better chance at accurately identifying a suspect by virtue of how much time they had to study the individual’s face, there is little the criminal justice system can do with that information. Conversely, system variables are those variables that affect eyewitness memory that the criminal justice system *can* control. As one might expect, the goal (generally) is to try to institute policies that maximize the likelihood of witnesses making a correct identification while minimizing false identifications. For example, the way in which a lineup is presented to a witness can impact their likelihood of success. Specifically, research suggests that simultaneous lineups result in superior witness identification performance relative to sequential lineups (e.g., Mickes et al., 2012; but see Horry et al., 2021 for a qualification).

The issue of lineup construction is among the most studied system variables. When an investigator is tasked with constructing a lineup, they are typically provided with (a) a verbal description of the perpetrator (i.e., the individual who actually committed the crime) by the eyewitness and (b) a photograph of the suspect (i.e., the individual identified as someone who could plausibly be the perpetrator). The investigator must then select several photos of people who are known to be innocent (i.e., fillers) to include in the lineup alongside the suspect. During the filler selection process, it is important to include fillers in such a way that the suspect does not “stand out”. That is, salient, identifiable features such as height and weight should be consistent across the lineup. If, for example, the suspect has a darker complexion than all the fillers, the suspect would unfairly stand out eliciting more identifications of both guilty suspects and, more concerningly, innocent suspects (Wells et al., 1993). Although the potential problems arising from fillers being too different is an intuitive one, researchers have identified that problems can exist along the other end of the continuum as well. Namely, when fillers appear *too* similar to the suspect (imagine the extreme hypothetical in which a suspect’s identical twin is included in the lineup), the task can become too difficult for eyewitnesses and correct identification rates suffer accordingly (Luus & Wells, 1991; see also Fitzgerald et al., 2015). Therefore, the lineup administrator should search for an optimized “sweet spot” of suspect-filler similarity that maximizes correct identifications while minimizing erroneous suspect identifications (Luus & Wells, 1991).

Although a great deal of research has examined the effect of suspect-filler similarity on identification performance (e.g., see Fitzgerald et al., 2013), these results are difficult to translate into practical advice for police procedure. For example, recent guidelines from the Department of Justice (DOJ) recommend selecting fillers that fit the general description of the offender, and are not too similar nor too dissimilar to the suspect (U.S. Department of Justice, 2017). However, it is unclear what “too similar” and “too dissimilar” actually mean. The DOJ goes further stating that the fillers “should be sufficiently similar so that a suspect’s photograph does not stand out, but not so similar that a person who knew the suspect would find it difficult to distinguish him or her.” Nevertheless, this recommendation still invites subjectivity, and therefore, its implementation will likely vary by individuals and precincts.

Perhaps because of the difficulty in defining similarity in a way that is more precise and objective, extant recommendations understandably focus on the *process* of filler selection rather than the specific desired *outcome* (Wells et al., 2000; see also Luus & Wells, 1991). This research argues that eyewitness identification is (generally) best served when the lineup is constructed with fillers matched to the witness’ description of the perpetrator rather than to the suspect themselves (Wells et al., 2000).

Although this recommendation provides concrete advice regarding filler selection, it is unclear whether a lineup with purely description-matched fillers will always lead to the optimal eyewitness performance (Tunnicliff & Clark, 2000). One problem with this recommendation is that it assumes a reasonably accurate and detailed description of the perpetrator (see Wells et al., 2020 on what to do when the description is inaccurate). However, there is evidence that witness descriptions are often missing important information, and that matching based on such incomplete descriptions can lead to elevated innocent suspect identification rates (Lindsay et al., 1994). In these cases, researchers recommend matching the fillers to the suspect on general characteristics, such as age, sex, and race (e.g., Wells et al., 2020).

In addition to the verbalizable features that witnesses sometimes omit, it is also important to consider the substantial category of features that do not easily lend themselves to being described. For example, there is a large body of evidence to suggest that face processing is holistic (e.g., Young et al., 2013), and therefore, not conducive to a feature-based process required to verbally describe a face (see also, Wells & Hryciw, 1984).

Further evidence arguing against the superiority of description-matched fillers are those studies which simply fail to demonstrate that such a strategy actually improves identification performance. For example, in two experiments, Tunnicliff and Clark (2000) compared the benefit of a lineup with description-matched fillers to a lineup with suspect-matched fillers. Despite using lineup constructors from different populations, performance was comparable between suspect-matched and description-matched lineups.^1^ Perhaps more convincingly, a recent meta-analysis examined how suspect-filler similarity affects identification performance (Fitzgerald et al., 2013). Collapsing across nine empirical studies, this analysis showed that lineups with fillers that were highly similar to the suspect yielded lower innocent suspect identification rates compared to a lineup with moderately similar fillers, but had no effect on correct identification rates.

Even if lineup administrators take the relatively straightforward advice of matching fillers to the witness’ description, ambiguity still persists after the features have been matched. That is, if a witness describes the perpetrator as a heavy-set, tall male in his late 40 s, once those features have been matched, how closely (if at all) should the fillers resemble the suspect? Current best practices suggest that fillers should be substantially dissimilar from the suspect after matching to the description (Wells et al., 1993; see also Colloff et al., 2021). This highlights that the recommendation to match fillers to the description of the offender does not eliminate the burden of considering suspect-filler similarity in any systematic way. Put differently, this suggests that some blended approach of filler selection yields better discriminability than using either approach in isolation.

Indeed, there are potential benefits and costs of both types of filler selection approaches. The benefit of selecting fillers based on a witness’ description is that it provides an obvious stopping point for how similar the fillers should be (e.g., if the witness mentions three physical characteristics of the perpetrator, officers can match on those three dimensions and nothing more), and reduces the amount of subjectivity involved in the selection process. However, the potential costs of using description-matched fillers are that descriptions can be inaccurate, or sparse in detail, which may partly stem from the fact that faces are difficult to describe (e.g., see Frowd et al., 2005; Meissner et al., 2007). In addition, as mentioned above, there is evidence that the level of similarity that optimizes performance is less similar than lineups with purely description-matched fillers (Colloff et al., 2021; Wells et al., 1993). Of course, selecting fillers based on the appearance of the suspect is more subjective and provides no obvious stopping point for how similar the fillers should be, which can lead to biased lineups. In the current study, we use a blended approach by matching fillers on general characteristics, and then further manipulating the similarity of the fillers to the suspect in a more objective fashion.

Looking broadly across the literature, generating clear recommendations about suspect-filler similarity is fraught because each study defines similarity idiosyncratically. Some researchers have attempted to approach operationalizing similarity by using morphing software which creates a new, artificial face from two or more seed faces. In this way, researchers can systematically vary how similar fillers are to the suspect by specifying exactly how much of the components of each seed are incorporated into the various composite faces. Using this approach, Fitzgerald et al. (2015) found that highly similar fillers yielded a lower correct identification rate compared to moderately similar fillers. This conclusion notably differs from these authors’ previous meta-analysis which indicated that the use of high-similarity fillers was not reliably associated with a reduction in correct identification rates. The authors speculated that the morphing software that was used allows for a much greater level of similarity than face photograph databases that are used by researchers. This suggests that the relationship between similarity and witness performance may be non-linear. That is, problems arise when fillers are both too dissimilar *and* too similar.

One may intuit, then, there is an ideal zone of similarity in which the fillers are neither too dissimilar nor similar that maximizes witnesses’ ability to make correct identifications. The critical question, then, is how do we define that zone? Historically, researchers have relied upon ordinal labels to characterize similarity, but that approach has led to the current ambiguous state reviewed thus far. A more objective approach to defining similarity would not only be beneficial for researchers comparing outcomes of various studies but also to policy makers and lineup administrators who could more easily apply prescriptive recommendations since those recommendations would not be reliant upon subjective decision making. The aforementioned face morphing software (e.g., Fitzgerald et al., 2015) might seem like a candidate solution due to its ability to objectively quantify how similar two or more faces are. However, as the authors noted, this procedure carries additional concerns of ecological validity. For example, this procedure requires one to use a relatively homogenous set of faces in order to yield fillers that do not appear to be morphs. Similarly, it is not obvious how identification performance is affected when all of the fillers are morphs of the suspect. This is because morphing would likely increase the familiarity of both the suspect and the fillers, and it is not clear that the increase would be comparable between the two types of photos. It is also possible that the morphing procedure would yield more typical fillers, as a result of the averaging among faces.

The current study aims to measure similarity in a more precise fashion, using multidimensional scaling (MDS, e.g., Kruskal, 1964a, b; Kruskal & Wish, 1978; Rabinowitz, 1975). MDS is an exploratory data analysis technique that provides a set of interitem distances in a *k*-dimensional space where *k* represents the number of dimensions that are specified by a given scaling solution. Importantly, the algorithm seeks to create a space in which perceived similarity is monotonically related to distance, among all the stimuli in the set. As a consequence, similarity can be measured such that stimuli are similar to the extent that they are closer in space (i.e., less distance). For example, imagine a hypothetical set of faces that vary on a number of dimensions, such as age, sex, eye size, etc. MDS attempts to determine which dimensions are most important in defining the similarity among the set of faces. In applying MDS to this hypothetical set of faces, one may find that two dimensions captures a sufficient amount of variation among the faces. Upon inspection of this face-space, one may notice that faces varying on one dimension vary in skin tone, whereas faces that vary on the other dimension vary in age. As a result, the researcher may infer that the two dimensions of the face-space are age and skin tone. MDS has been quite useful in measuring psychological similarity (e.g., Clark et al., 1986; Hout et al., 2016; Howard & Howard, 1977; Papesh & Goldinger, 2010; Shepard, 1980), particularly because it allows researchers to infer the specific dimensions by which the space is defined. As a result, this gives researchers some idea as to which dimensions are most important in defining similarity.

One limitation of MDS is that it often relies on data collection that is time-consuming and inefficient, as it requires participants to make pairwise comparisons among all possible pairs of stimuli (but see Goldstone, 1994; Hout et al., 2013 for alternative data collection methods). That is, for a stimulus set of *n* items, *n*(*n* − 1)/2 ratings are required. As such, a database of, for example, 100 faces necessitates 4950 ratings (Goldstone, 1994). Because the number of comparisons required can grow quite rapidly, it is impractical to solicit ratings for anything but small face databases.

Fortunately, human responses are not required to create an MDS face space; there are other approaches. Specifically, faces can alternatively be quantified by measuring the distance (using computer software) between specific landmarks of a given face (e.g., tip of the nose, the corners of the eyes and mouth, etc.). With these measurements, each face can be defined as a vector of numbers, which can be used to compute a measure of Euclidean distance for each pair of faces. These distances can then be used to construct an *n*-dimensional “face-space” in a similar fashion as described earlier (albeit without the labor associated with gathering human-provided responses). By employing these objective measurements, we can circumvent the issues reviewed previously concerning the difficulties in operationalizing similarity. We should note that this approach it is not completely objective in that it does not completely remove human judgment from the process. This is because researchers and investigators have some discretion over the amount and type of data that each face contributes. Nevertheless, we think that this approach provides a more objective way of defining similarity than much of the previous research.

Using such an approach, we are able to use MDS to create a face space from a database of faces far larger than what would be possible using human ratings. In the proposed study, we intend to use a set of 82,028 mugshots from which we extracted information about each face. From each face we extracted information such as age, sex, and race but the majority of the information extracted relates to facial landmarks (referred to as fiducials, see Materials for more information). Using a database of this size is critical because it provides us with a large sample of photos to choose from, which should allow us to find fillers that are *exceptionally* similar to the suspect. Some have suggested that because investigators often have access to a much larger database of photos to select fillers from, relative to researchers, studies may not be observing this relationship at the higher end of the similarity scale (Bergold & Heaton, 2018; Fitzgerald et al., 2013, 2015). Therefore, a database of this size affords us more precise control over the degree of suspect-filler similarity. In addition, a database of this size will yield an MDS space that is more representative of how faces vary.

To the extent that our method of quantifying similarity accurately captures how faces are perceived (see Tredoux, 2002), this approach will be useful in more precisely operationalizing similarity, which should prove useful in theory development and testing. For example, the recommendation to select fillers based on the description of the offender (Carlson, et al., 2019; Colloff et al., 2021; Juslin et al., 1996; Lindsay & Wells, 1980; Luus & Wells, 1991; Navon, 1992; Technical Working Group, 2003; U.S. Department of Justice, 2017; Wells et al., 1993) was, in part, based on the notion that description-matched fillers will lead to lineups with “propitious heterogeneity” among lineup members (Luus & Wells, 1991), which should aid recognition (Gibson, 1969). Indeed, subsequent research found that dissimilar fillers that are otherwise matched to the description of the offender enhanced identification performance relative to suspect-matched lineups (Wells et al., 1993). More recently, the diagnostic-feature-detection (DFD) theory was introduced (Wixted & Mickes, 2014), stating that the discriminability of a procedure is determined by the extent to which it emphasizes which features are diagnostic. For example, when fillers are matched to the features in the description of the offender, these features are rendered nondiagnostic, which allows a witness to focus on the more diagnostic features. Subsequent modelling (Colloff et al., 2021) showed that the DFD theory predicts a benefit of dissimilar, description-matched fillers over more similar fillers, as Luus and Wells (1991) predicted. They also confirmed this prediction empirically by replicating Wells et al. (1993). Therefore, in the current study, we should find that lineups with the least similar fillers, within a given subset of mugshots generally matching on age, sex, and race, should yield the greatest discriminability. That is, in the current study, both the propitious heterogeneity and the DFD hypotheses predict that discriminability will be greatest for lineups with fillers that are on the lower end of the similarity scale, after matching on the general characteristics of the offender.

In the proposed experiment, participants will be shown a series of faces to study. After initial encoding, they will complete a distractor task, followed by four lineups with the studied face (“guilty suspect”) or an unstudied face (“innocent suspect”) among 5 fillers. Each lineup will be associated with only one of the studied faces. Importantly, we will vary the similarity between the suspect and filler. This design allows us to examine how suspect-filler similarity affects discriminability across a wide range of the similarity scale.

## Method

### Participants

A power analysis was conducted using the powe(R)OC app in R (Mah, 2022). This analysis indicated that 400 participants are required in order to detect an effect size of 0.15 with approximately 0.8 power. This effect size is equal to $1-\frac{{\mathrm{pAUC}}_{1}}{{\mathrm{pAUC}}_{2}}$ where ${\mathrm{pAUC}}_{1}$ is greater than or equal to ${\mathrm{pAUC}}_{2}$. This effect size value is based on the comparison between high-similarity fillers and low-similarity fillers from Colloff et al. (2021, Exp. 1). Participants will be recruited from either CloudResearch or Prolific to participate in this study. The online platform that we use will depend on the cost of the study, as well as considerations of which platform has been reported as yielding better data quality. We will aim for a total of 400 participants (before applying the exclusion criteria). All of the participants will be 18–60 years old. Any participants who fail the attention check will be excluded. Specifically, participants will be excluded if they exit full screen, or click outside of the browser running the experiment. In addition, we will include a simple prompt that requires a text-based response. Specifically, we will ask them: “Please describe a past experience that you enjoyed.” Participants who do not provide a sensical response to this prompt will also be excluded. All of the data will be stored on the first author’s OSF page.

### Design

A 4 (Suspect-filler similarity: High, Medium–High, Medium–Low, Low) $\times$ 2 (Target presence: Present, Absent) within-subjects design will be used in this study. There will only be one lineup per studied face. There will be a total of six blocks. For each block, there will be four sequential study trials. In each trial, one face will be studied. After a distractor task, there will be four lineup trials. For each lineup trial, one of the four faces will be tested on. Race and Sex will be random variables with the constraint that each are represented in each block. Suspect-filler similarity and target presence will be balanced across all six blocks.

### Materials

**Fig. 1:**
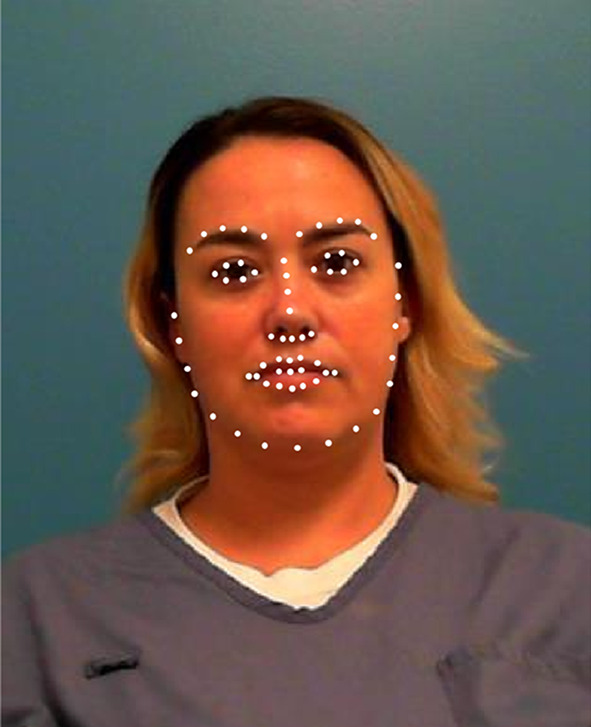
One face with their facial landmarks overlayed on their face in white dots. The *x*- and *y*-coordinates of the face as used in the MDS solution, along with: their actual age, their predicted age, numbers corresponding to the prediction of how likely the face belongs to each of the following races (i.e., Asian, Indian, Black, White, Middle Eastern, and Hispanic). *Note*: the actual landmarks have been increased from their pixel-length diameter for viewing purposes. There are 68 points but some of the points overlap, giving the appearance of only 64

We downloaded 82,028 mugshots from the publicly available offender database maintained by the Florida Department of Corrections (FL DoC; http://www.dc.state.fl.us/). We also downloaded information about the age of each offender at the time in which the mugshot was created, as well as the sex and race of each offender. From these we used the mugshots of offenders at least 20 years of age, but no older than 49 years of age. Finally, we only used black and white males and females (the lower quantity of faces in other racial categories and the lack of information about non-binary gender precluded a broader examination). In order to ensure that the resolution and size of all of the pictures were similar, we only used pictures that were at least 380 pixels in length and had an aspect ratio of at least 1.2. In doing so, there were 1116 black females, 27,535 black males, 2369 white females, and 22,425 white males. For each of these images, we used OpenCV (Bradski, 2000) to extract facial landmarks, as well as make predictions about the person’s age, sex, race, and emotion. We included only facial landmarks, the person’s predicted age, and race because the program did a poor job at predicting sex and this was not expected to vary within a subset of mugshots. We also did not include information on emotion because this characteristic is not inherent to the person’s appearance. For each image, we used 136 facial landmarks (i.e., 68 pairs), their actual age, their predicted age, and five values, each pertaining to the probability that a given face is Asian, Indian, Black, Middle Eastern, White, and Latin (see Fig. 1 for an example). These sets were further divided into three bins: individuals aged 20–29 years old, 30–39 years old, and 40–49 years old. This was done for all of the categories. Table 1 shows the frequencies of each set. For each subgroup, we will create a 10-dimenisonal space. There is no strong consensus on how many dimensions should ideally be implemented, as researchers have argued that this number is anywhere from three to 70 (see Lewis, 2004). Some studies have argued for a lower range (three to six dimensions; Busey, 1998; Lee, et al., 2000; Rhodes, 1988; Steyvers & Busey, 2000), whereas others arguing for a much larger range (10+; see Lewis, 2004 for a discussion). Our decision to use ten dimensions was based on a desire to specify a dimensionality that led to a maximal reduction in error variance, but also yielded solutions that fit each subgroup similarly.

**Table 1:** Frequencies of mugshots used, as a function of race, sex, and age group

Race	Sex	Age group			Total
		20–29	30–39	40–49	
Black	Males	8673	11,248	7614	27,535
	Females	396	441	279	1116
White	Males	4999	9652	7774	22,425
	Females	514	1101	754	2369
	Total	14,582	22,442	16,421	53,445

**Fig. 2:**
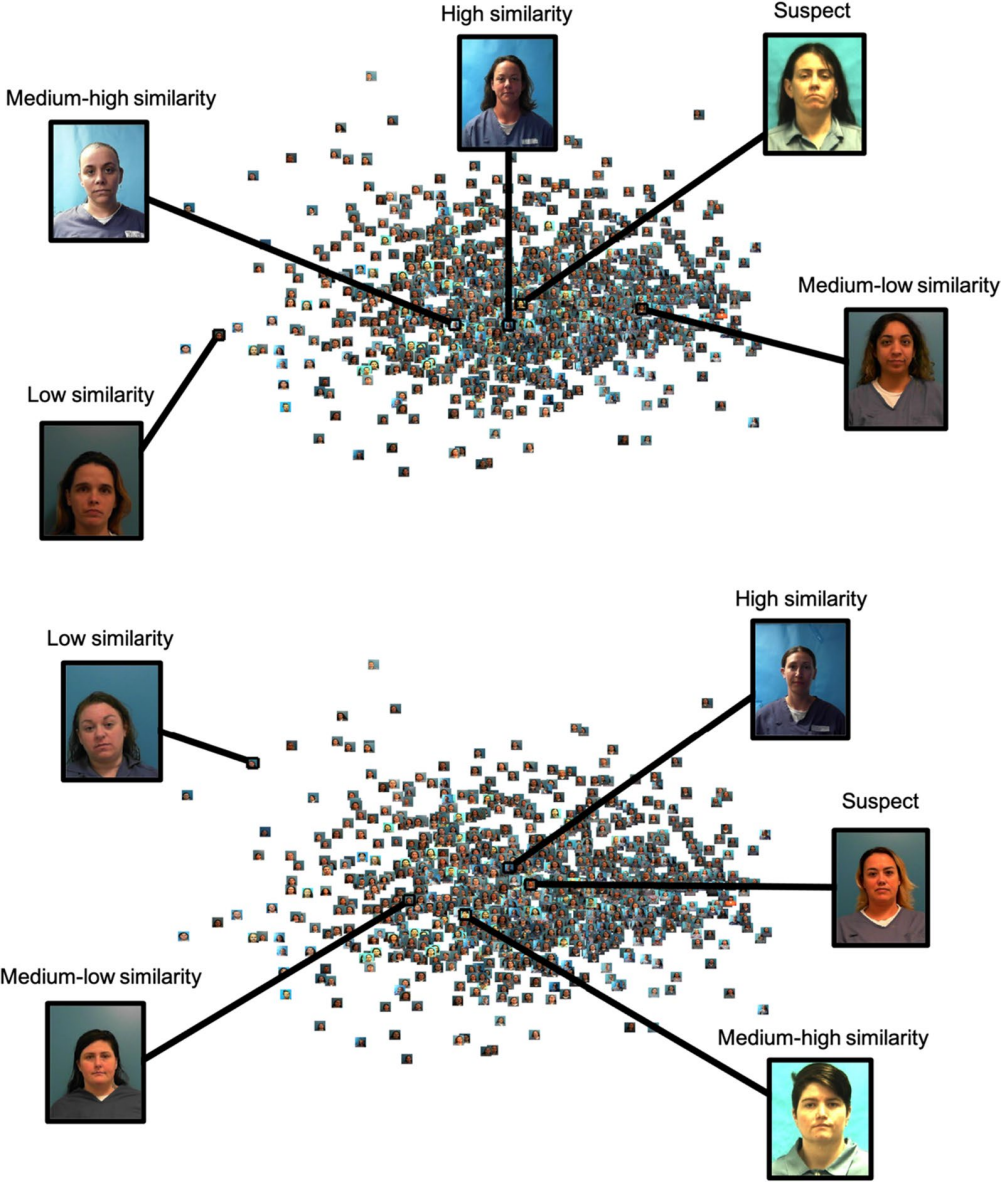
A two-dimensional face space of white females in their 30 s. Each panel shows an example of a suspect, with a potential filler for each level of similarity, based on distance from the suspect. The top panel shows an example using a randomly determined guilty suspect. The bottom panel shows an example using a randomly determined innocent suspect. Note: Two dimensions were specified for these spaces for illustrative purposes only

For each participant, we will create 24 lineups. For each target-absent (TA) trial, we will randomly choose two images. One of these images would be studied, and one would serve as the innocent suspect. For each target-present (TP) trial, we will randomly choose only one image which would be studied and included in the subsequent lineup as a guilty suspect. For each set of four lineups, we will choose images from a randomly selected age group, for each sex by race combination, without replacement. For each trial, the fillers for each lineup will be selected based on their similarity to the suspect (i.e., their distance from the suspect in the MDS space). In the high-similarity trials, the five photos closest to the suspect will be selected to be fillers. In the medium–high similarity trials, photos that are closer to the suspect than around 66% of the photos will be selected to be the fillers. In the medium–low similarity trials, photos that are closer to the suspect than around 33% of the photos will be selected to be the fillers. Finally, in the low-similarity trial, the five photos that are the farthest from the suspect will be chosen to be the fillers (see Fig. 2). For subsequent trials within a given sub-group, all of the used photos will be removed from the pool of available mugshots prior to selecting fillers. Across all 24 lineups, there will be an equal number of lineups from each race by sex combination. Each study and lineup trial will be randomly assigned to one of six blocks with the constraint that each sex by race combination is represented once.^2^

### Procedure

Participants will study six blocks. For each block, four photos will be presented sequentially for 5 s each.^3^ Each block will contain one Black female, one Black man, one White woman, and one White man. After studying all four photos, participants will be given a distractor task (i.e., Tetris) for 60 s. Following this task, participants will be given four lineups, one after the other. For each lineup, they will be asked “Is the white (or black) woman (or man) from the last four photos present in the lineup?”. They will further be told that the person from study may or may not be present in the lineup, and to select them if they are present, and reject the lineup if they are not present in the lineup. After each response, they will then be asked to give a confidence rating in their decision, on a scale from 0 to 100. For each block, the order of test position will match the order the study position. The order of study position and test position will be randomized for each block. The experiment can be viewed at the following link: https://jspsych.hrumc.domains.skidmore.edu/gmckinley/jspsych-6.1.0/examples/FoilSim_exp_testing.html.

### Proposed analyses

#### Discriminability

Our main question is how suspect-filler similarity affects discriminability. We plan to perform two analyses to answer this question. For each analysis, we will first compare whether the two medium-similarity conditions differ from each other. If they do not differ, we will collapse the data from these two conditions, in order to reduce the number of pairwise comparisons from six to four. A Bonferroni correction will be applied. Because we are predicting that fillers that are less similar to the suspect will do better than fillers that are more similar, each comparison will be a one-tailed test, with the lineup with less similar fillers performing better than the lineup with more similar fillers. For all analyses, we will use R (R Core Team, 2020). First, we plan to conduct ROC analysis, using the pROC package (Robin et al., 2011) and compare *partial area under the curve* (pAUC) between suspect-filler similarity conditions. Each curve will plot 11 hit rate (HR) and false alarm rate (FAR) pairs over decreasing levels of confidence. The first HR-FAR pair will correspond to cases in which participants respond with 100% confidence. The second HR-FAR pair will correspond to cases in which participants respond with 90% or more confidence. This will continue up to the rightmost points of the curves which will correspond to cases in which participants choose a suspect with any confidence. For statistically comparing the pAUCs of each condition, we will truncate the ROCs to the condition will the lowest cumulative false alarm rate. We will also fit signal detection theory models (both the equal-variance and unequal-variance versions) to the data and compare the three (or four) conditions to each other in terms of d' (in the unequal variance case) and ${\mu }_{T}$ (in the equal variance condition). For this, we will use the sdtlu package (Cohen et al., 2021). In order to reduce the number of parameters that need to be estimated, we will combine confidence ratings of 0–20, 30–40, 50–60, 70–80, and 90–100 into a 5-point confidence scale. If this 5-point confidence scale yields any cells with fewer than 5 observations, we will use a 3-point confidence scale (i.e., 0–60, 70–80, and 90–100). For each participant, there will only be three observations per target-presence and similarity combinations. Therefore, all analyses will be done on the disaggregated data. That is, pAUC and the model fits will not be computed for each participant, and the data will be treated as though each observation is independent from each other. For both the ROC analysis and the model fits, the false alarms will be based on instances in which the designated innocent suspect is chosen.

For exploratory purposes, we will also repeat all of the analyses above for each Race X Sex combination. This will be done because the number of mugshots in each subgroup differs substantially. For example, there are more than twice as many white females as there are black females, and there are more than fourteen times more males than females. Therefore, it is quite likely that a greater level of similarity can be achieved when lineups are constructed when sampling from a larger number of mugshots (Bergold & Heaton, 2018).

#### Confidence-accuracy calibration curves

Although it is not our focus, we plan to construct confidence-accuracy calibration curves for each condition to examine whether suspect-filler similarity affects the confidence-accuracy relationship. This analysis would be purely exploratory. For each similarity condition, the correct suspect identification rate [i.e., correct suspect identifications/(correct suspect identifications + incorrect suspect identifications)] will be computed for a confidence of 0–60, 70–80, and 90–100. A bootstrapping procedure will be used to compute the standard error of a given difference, which will allow us to statistically compare how confidence affects accuracy within each condition.

## Data Availability

All of the data will be available on OSF once data collection has finished. Stimuli will not be provided due the potential ethical issues of increasing the accessibility of mugshots of prisoners.

## Footnotes

1 It is worth mentioning that in both experiments, discriminability, as measured by d' showed an advantage for suspect-matched lineups, though, the methods were not statistically compared using this measure.

2 2 Across all blocks, suspect-filler similarity will be crossed with target presence. However, block will not be crossed with either of these variables, and neither of these variables will be crossed with race and sex.

3 3 The experiment will be piloted on a few participants to determine if this duration is sufficient or not. If performance is near floor, the duration will be increased to no more than 10 s. The duration will be decreased to 3 s if performance is at ceiling.
